# Supplementary material for: Hox genes regulate asexual reproductive behavior and tissue segmentation in adult animals
Source: Nat Commun. 2021 Nov 18;12:6706. doi: 10.1038/s41467-021-26986-2 (PMC8602322; doi:10.1038/s41467-021-26986-2)
Supplement: Supplementary file 1 — Supplementary Information [file 41467_2021_26986_MOESM1_ESM.pdf]

Supplementary Materials for

Hox genes regulate asexual reproductive behavior and tissue segmentation in adult animals

Authors: Christopher P. Arnold[1], Analí Migueles Lozano[2], Frederick G. Mann, Jr.[1], Stephanie H. Nowotarski[1], Julianna O. Haug[1], Jeffrey J. Lange[1], Chris W. Seidel[1], Alejandro Sánchez Alvarado[1,3,*]

Affiliations:

[1]Stowers Institute, USA; [2]University of Chicago, USA; [3]HHMI, USA

* Correspondence to: asa@stowers.org

This PDF file includes:

Supplementary Figures 1-11

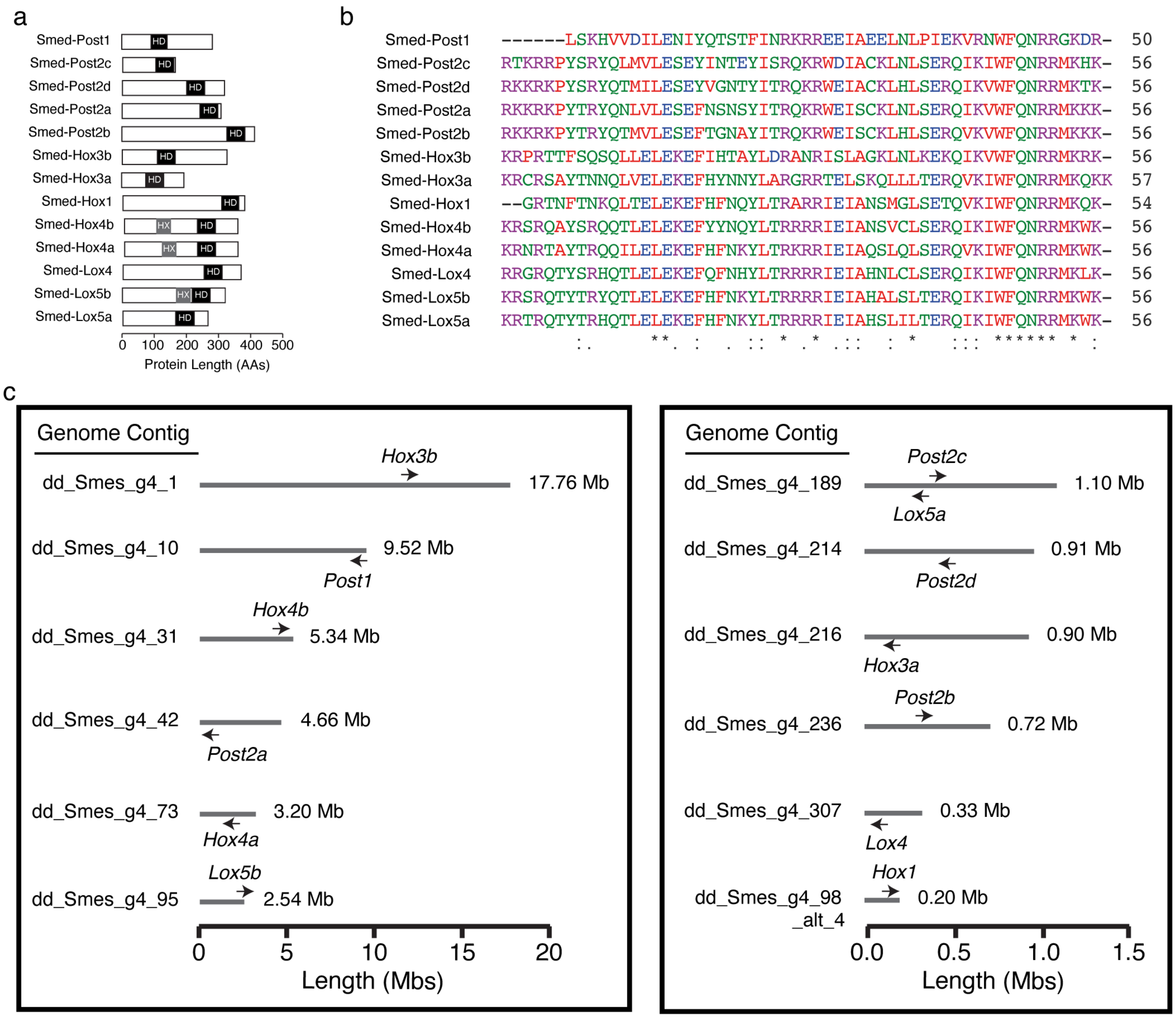

Supplementary Figure 1. Hox genes of the planaria *Schmidtea mediterranea.*

a Relative size, hexapeptide motif (HX) location, and conserved homeodomain location (HD) of the 13 planarian Hox family proteins of *Schmidtea mediterranea*. **b** Sequence alignment of the homeodomains of the 13 planarian Hox family proteins. **c** Diagram of the genomic distribution, location, and directionality of the 13 planarian Hox family genes.

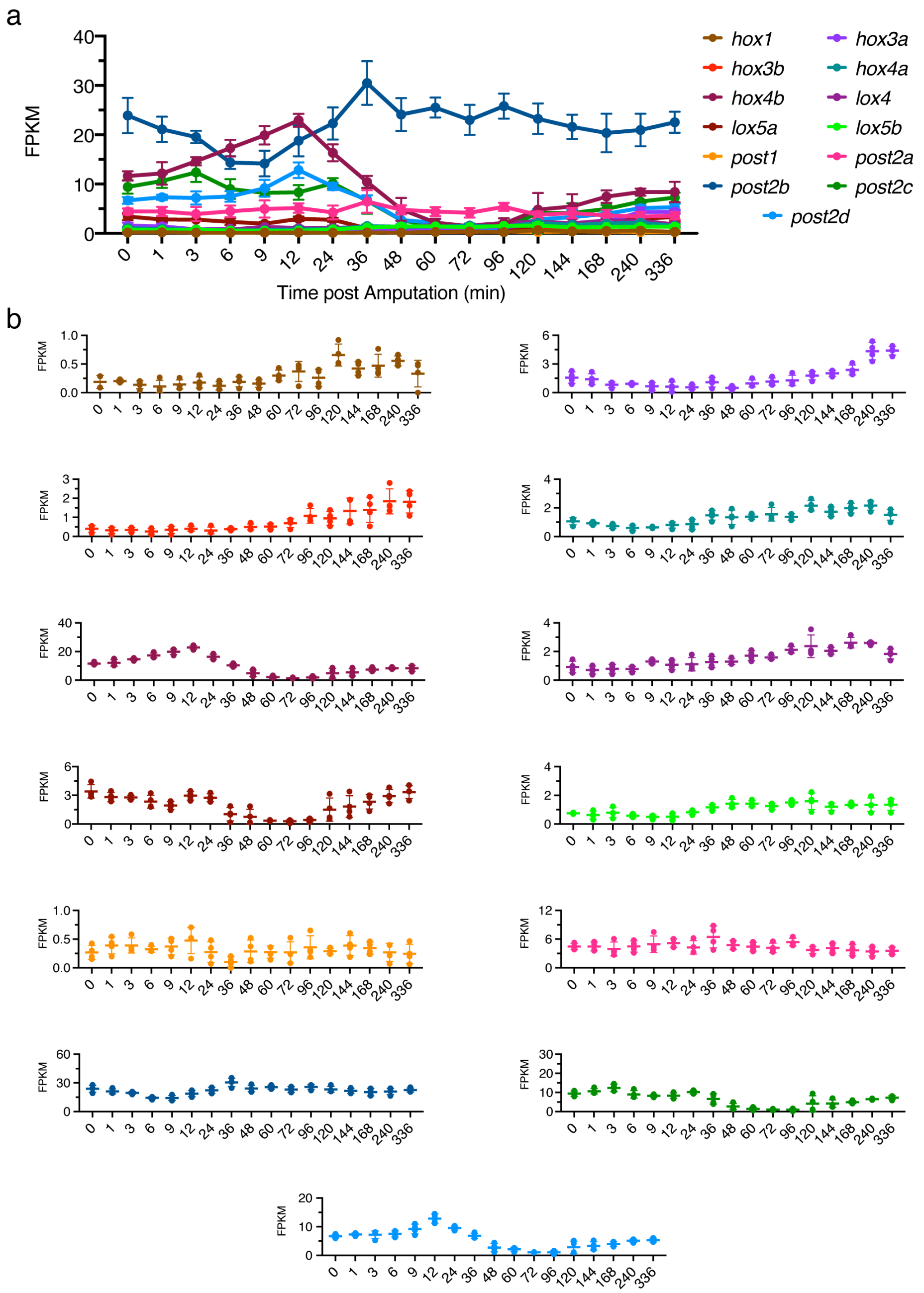

Supplementary Figure 2. Hox gene expression dynamics during planarian regeneration*.*

a-b Time courses of the mRNA expression of (**a**) all 13 Ho*x* genes or (**b**) each Hox gene during the regeneration of laterally amputated tissue fragments. Expression levels plotted as FPKM (n= 3 biological replicates, mean and SD) from RNAseq analysis. Data sourced from Zeng et al., 2018.

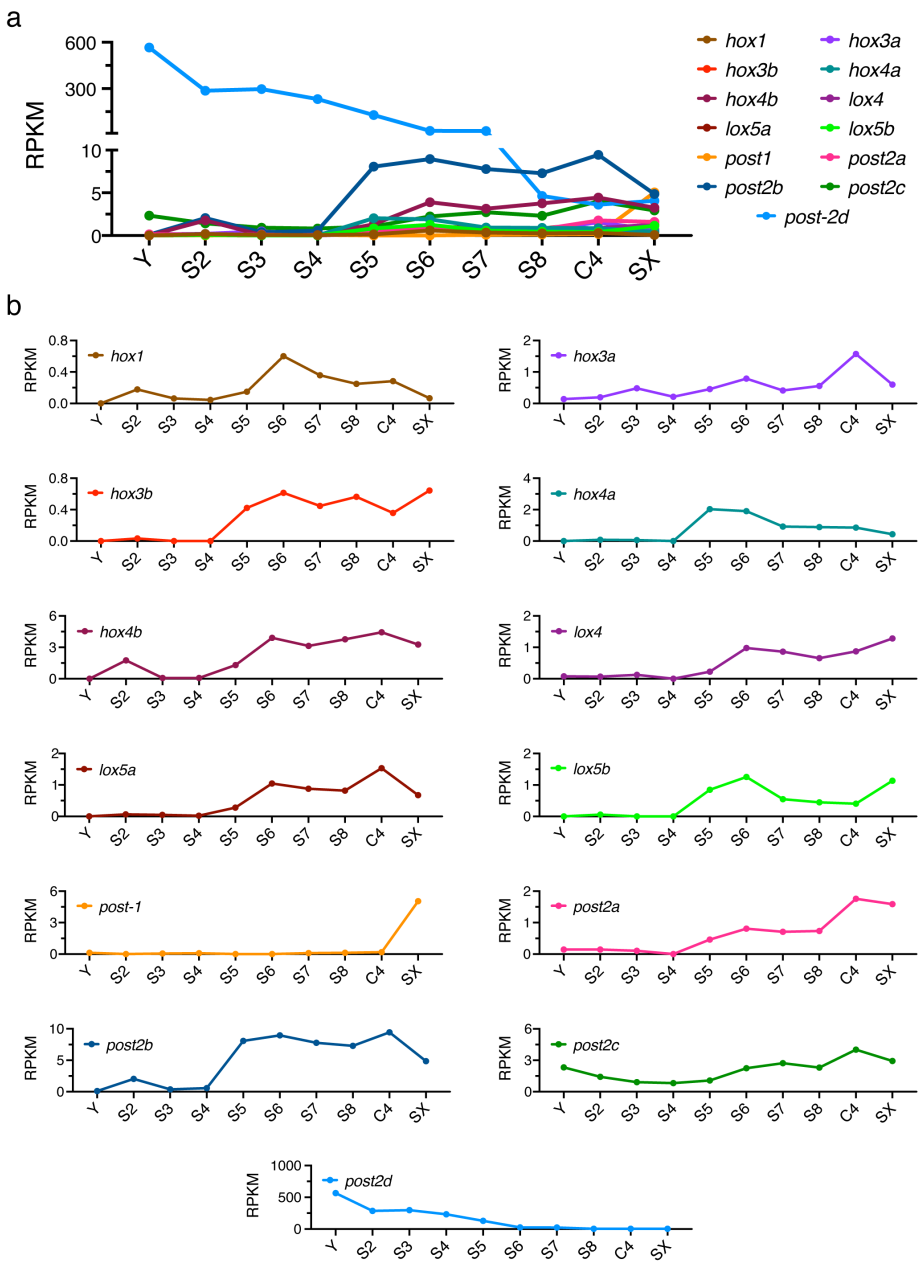

Supplementary Figure 3. Hox gene expression dynamics from planarian embryogenesis to adulthood.

a-b Time courses of the mRNA expression of (**a**) all 13 Hox genes or (**b**) each Hox gene at each stage of embryogenesis of the sexual strain and the adult stage of the sexual and asexual strains. Expression levels plotted as RPKM from RNAseq analysis (Y= egg yolk, S2-S8 = embryonic stages 2-8, C4= adult asexual planaria, SX= adult sexual planaria). Data sourced from Davies et al., 2017.

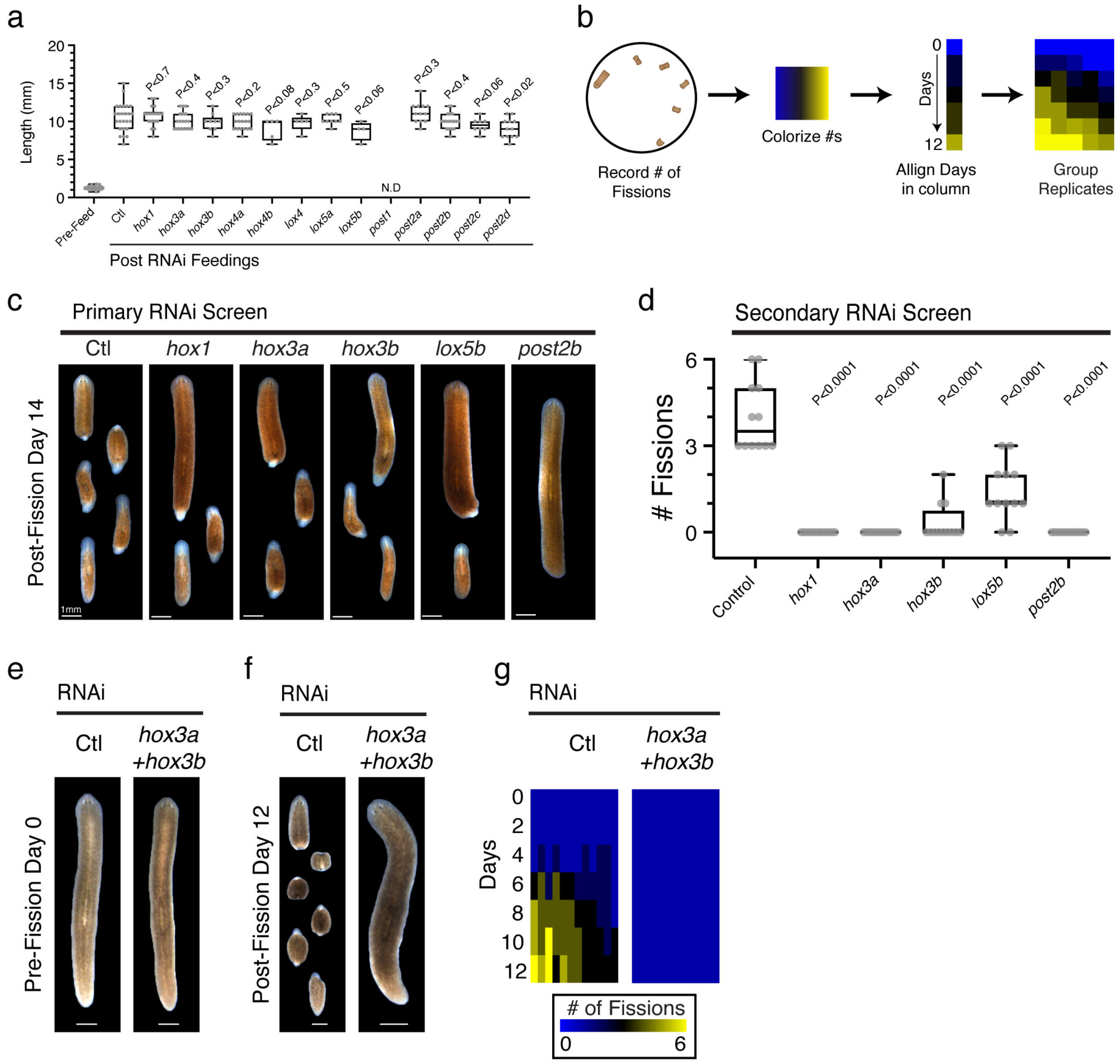

Supplementary Figure 4. A subset of planarian Hox genes regulate fission

a Box plots of size distribution of planaria prior to and following nine purified dsRNA feedings targeting Hox genes (n=25, 18, 18, 9, 18, 5, 5, 9, 4, 12, 18, 8, and 12 animals for Ctl, *hox1*, *hox3a*, *hox3b*, *hox4a*, *hox4b*, *lox4*, *lox5a*, *lox5b*, *post2a*, *post2b*, *post2c*, *post2d* RNAi samples, respectively; Ctl vs. *hox1*, *hox3a*, *hox3b*, *hox4a*, *hox4b*, *lox4*, *lox5a*, *lox5b*, *post2a*, *post2b*, *post2c*, *post2d* p-values=0.681, 0.3793, 0.2545, 0.158, 0.0766, 0.2715, 0.4992, 0.0565, 0.2389, 0.3081, 0.051, and 0.0114, respectively; animals that fissioned prior to experimental setup were omitted from size measurement since they

were no longer intact). Source data are provided as a Source Data file. **b** Diagram of data visualization. The number of cumulative fissions for each day was converted to a heat color code. Daily fissions for each worm were aligned in ascending order along the y-axis. The average score of each column is calculated and used to sort individual worms in descending order along the x-axis. The result is a heatmap visualization of fission activity across replicates. **c** Representative images of animals from primary Hox RNAi screen on day 14 of the fission assay (n= 18 animals). **d** Plot depicts fission progeny number on day 12 of secondary RNAi screen (n=12 animals; Control vs. each Hox RNAi p-value<0.0001; 3 independent repeats). Source data are provided as a Source Data file. **e-f** Representative images of animals of control and *hox3a+hox3b* RNAi animals (**e**) prior to and (**f**) 12 days after fission induction (n=12 animals, 17 bacterial RNAi feedings, 3 independent repeats). **g** Heatmaps depicting cumulative fissions over time for individual worms following control and *hox3a +hox3b* RNAi-treatment (n=12 animals). Box and whisker plot depicts individual data points, median (centre), $25^{th}/75^{th}$ percentile (bounds of box), and minima/maxima (whiskers). P-value calculated by Welch's two-tailed t-test versus corresponding control. Source data are provided as a Source Data file. Scale, 1mm. Note: Control RNAi animals from **e-g** are from the same experiment as Fig. 1 e-f.

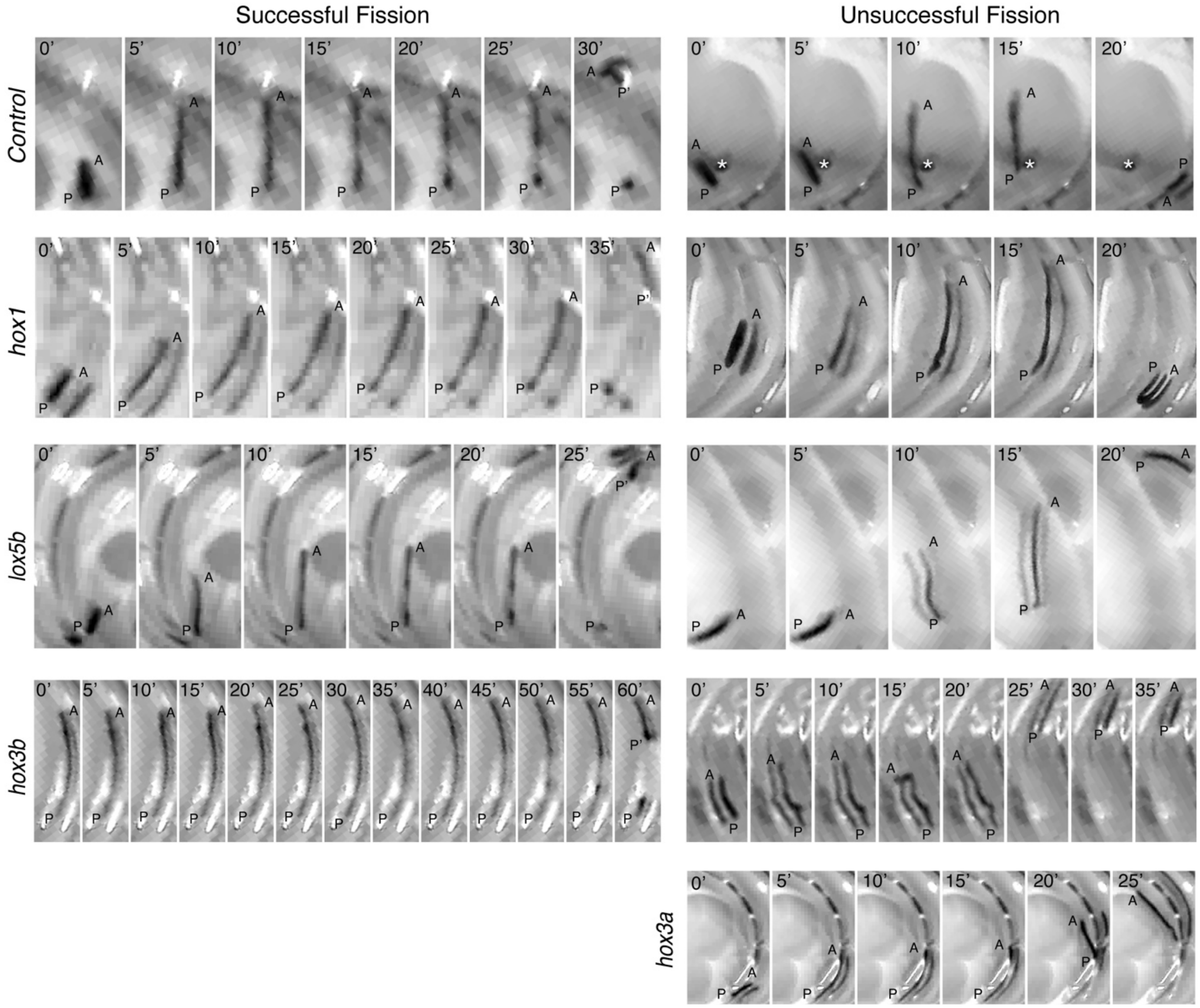

Supplementary Figure 5. Time-lapse Images of Successful and Unsuccessful fissions following Hox gene RNAi-treatment.

Representative time courses of fission attempts in RNAi-treated animals (n=5-6 animals, A= anterior, P=pre-fission posterior, and P'=post-fission posterior). Animals fed purified dsRNA food eight times.

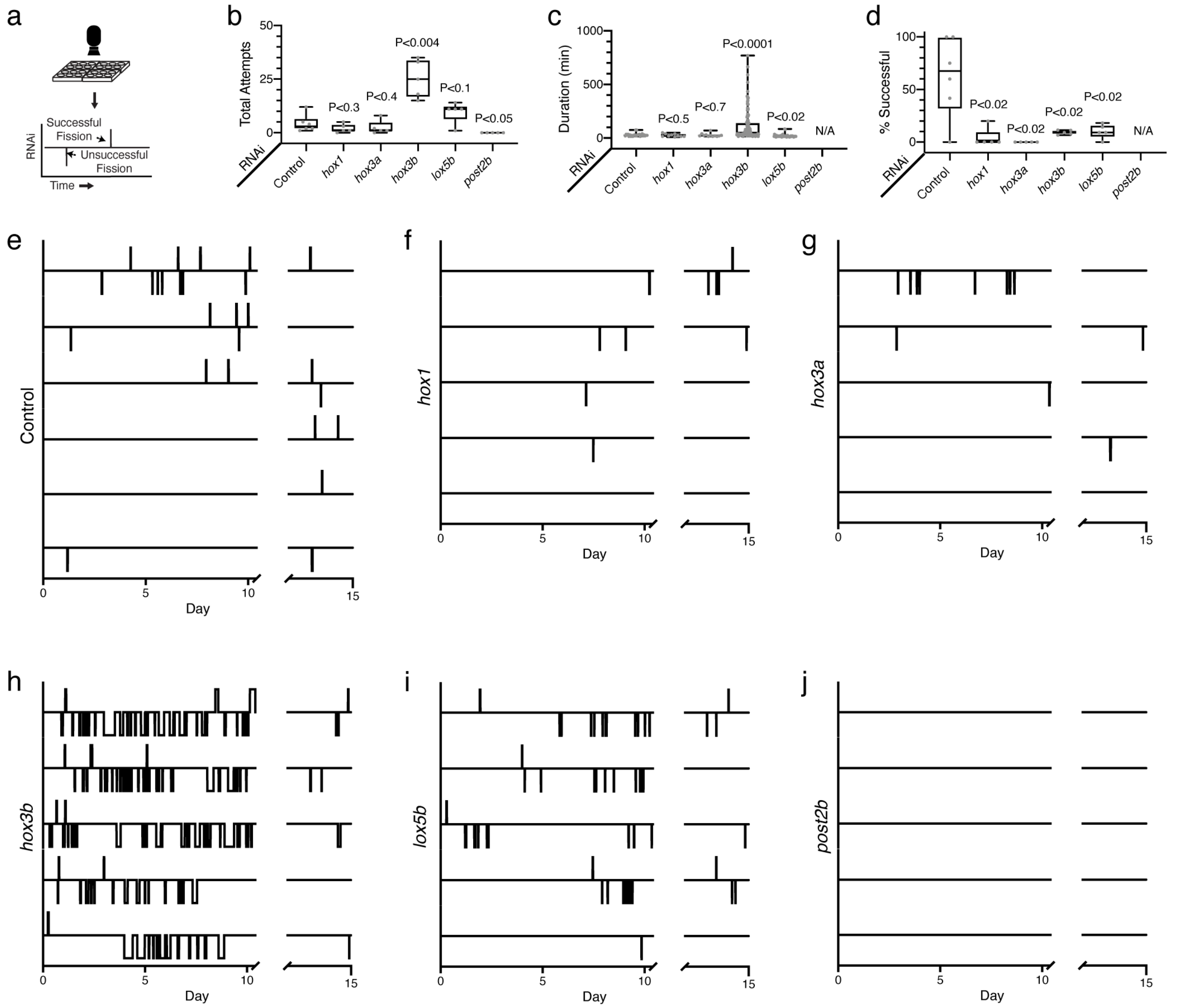

Supplementary Figure 6. Hox genes regulate the frequency, duration, and success of fission behavior.

a Schematic of webcam live imaging data visualization. Timeline depicts successful (upward displacement) and unsuccessful fissions (downward displacements). **b-d** Plots of the (**b**) total (Control vs. *hox1*, *hox3a*, *hox3b*, *lox5b*, and *post2b* p-values=0.2502, 0.3991, 0.0038, 0.0951, and 0.0464, respectively), (**c**) duration of (Control vs. *hox1*, *hox3a*, *hox3b*, *lox5b*, and *post2b* p-values=0.4851, 0.6368, 0.0038, <0.0001, and 0.0147, respectively), and (**d**) percentage of successful fission attempts (Control vs. *hox1*, *hox3a*, *hox3b*, and *lox5b*, p-values=0.012, 0.0101, 0.0186, and 0.0191, respectively) following Hox RNAi-treatment (n=6 and 5 animals for Ctl and all other RNAi samples, respectively;

p-value calculated by Welch's two-tailed t-test versus control). **e-j** fission activity timelines of (**e**) control, (**f**) *hox1*, (**g**) *hox3a*, (**h**) *hox3b*, (**i**) *lox5b*, and (**j**) *post2b* RNAi-treated animals. Animals fed purified dsRNA food eight times. Discontinuity in timeline due to a gap in image acquisition from technical error. Box and whisker plot depicts individual data points, median (centre), 25th/75th percentile (bounds of box), and minima/maxima (whiskers). Source data are provided as a Source Data file.

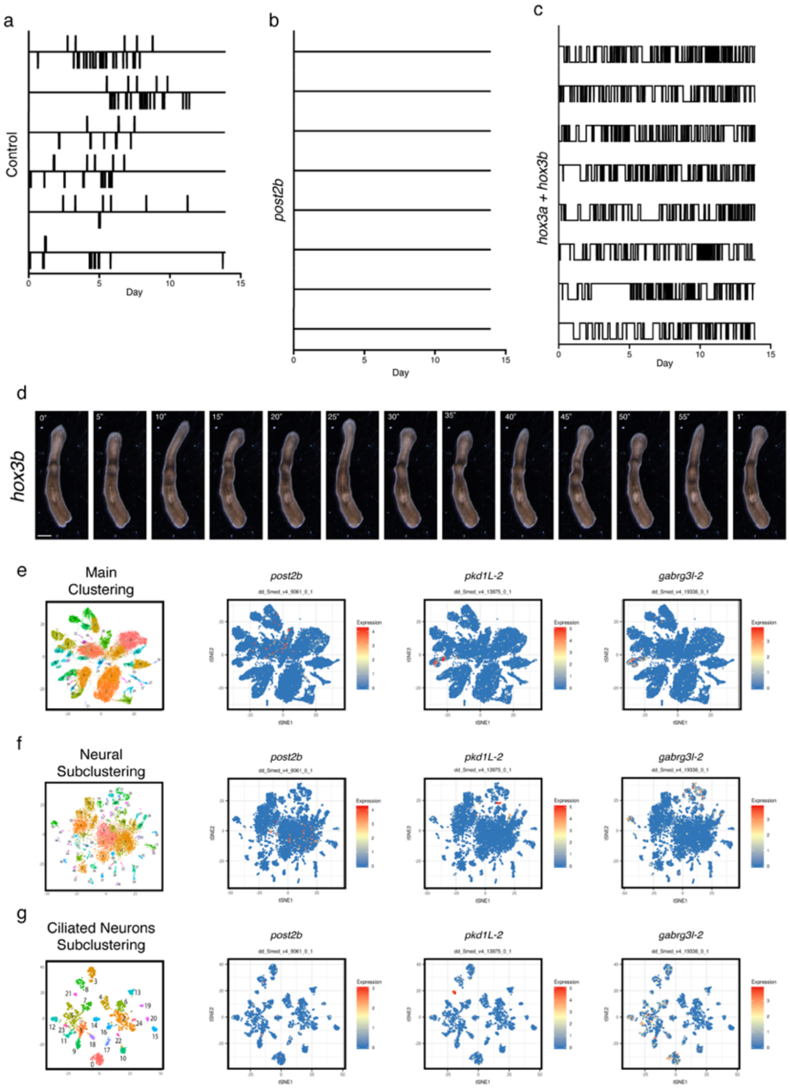

Supplementary Figure 7. *post2b* and *hox3* mediate opposing regulation of fission behavior.

a-c Fission activity timelines of (**a**) control, (**b**) *post2b*, (**c**) *hox3a+hox3b* RNAi-treated animals (n=6-8 animals). Source data are provided as a Source Data file. **d** Representative images of *hox3b* RNAi-treated animals stalled in Phase 1 (n=3/10 animals). Animals fed bacterial RNAi food 17 times. Scale, 1mm. **e-g** t-SNE plots of *post2b, pkd1L-2, and gabrg3l-2* data from (**a**) main clustering, (**b**) neural sub clustering (**c**) ciliated neurons sub clustering from the cell type transcriptome atlas (Data sourced from Fincher et al., digiworm.mit.edu)

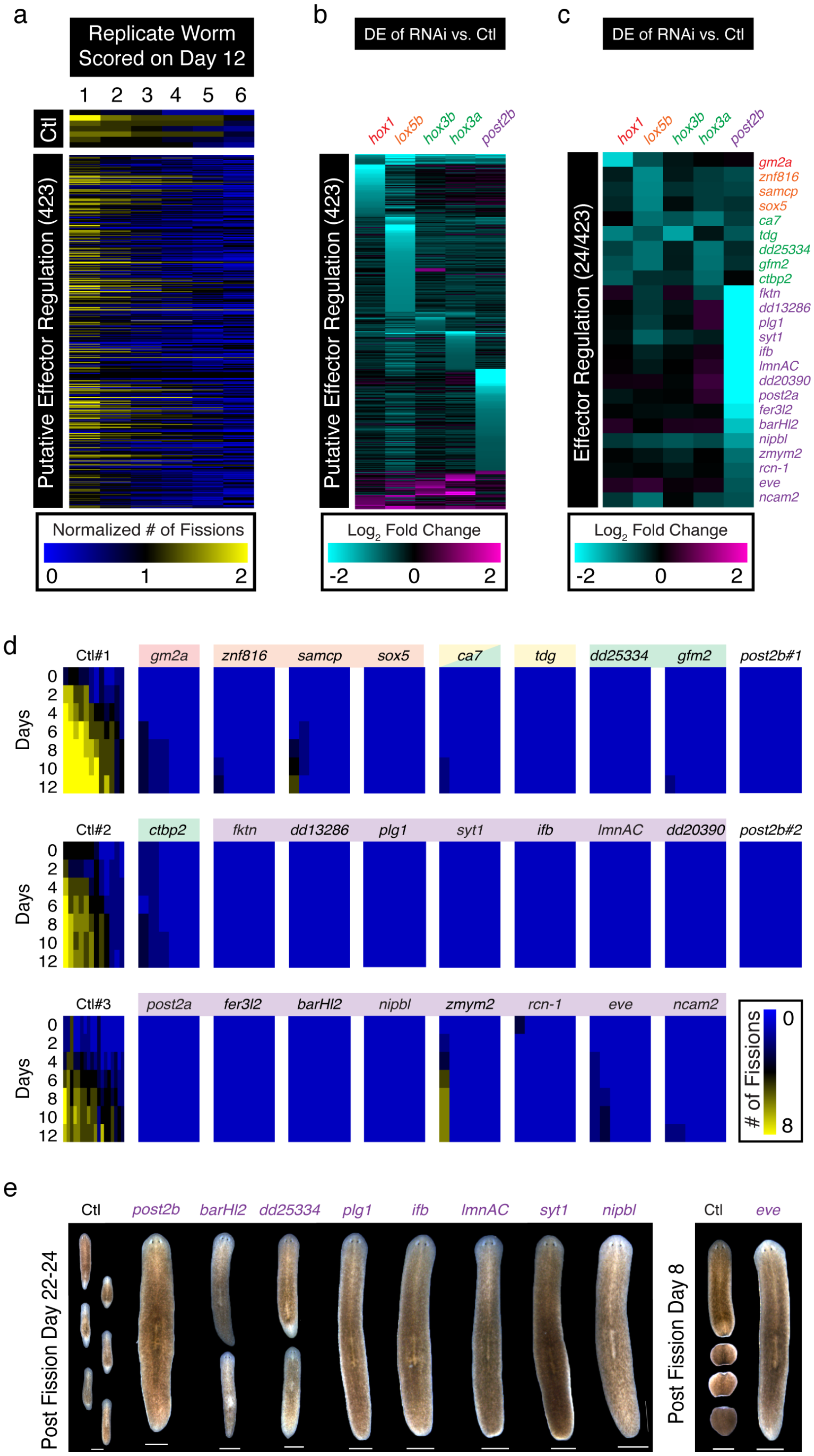

Supplementary Figure 8. A screen for downstream effectors of Hox genes.

a Heatmap depicting resultant fission activity following RNAi individually targeted against 423 putative effector genes from the DEGs of the Hox RNAi RNAseq (n=21 animals for

Control RNAi condition and 6 animals for each putative effector RNAi condition, fed 16-19 times with bacterial RNAi food). Heatmap depicts normalized fission number on Day 12 across six RNAi-treated worms. Source data are provided as a Source Data file. **b** Heatmap of the corresponding DE of each of the 423 putative fission effectors following RNAi of Hox genes. **c** Heatmap of the corresponding DE of each of the 24 fission effectors following RNAi of Hox genes. **d** Heatmaps of the number of fissions over time following RNAi of fission effectors (n=6-18 animals). Source data are provided as a Source Data file. **e** Representative images of *post2b* effector RNAi-treated animals 22-24 days or eight days post fission induction (2 independent repeats). Effector genes are color coded with respect to their putative upstream Hox gene: *hox1* (red), *lox5b* (orange), *hox3* (green), *post2b* (purple). Scale, 1mm.

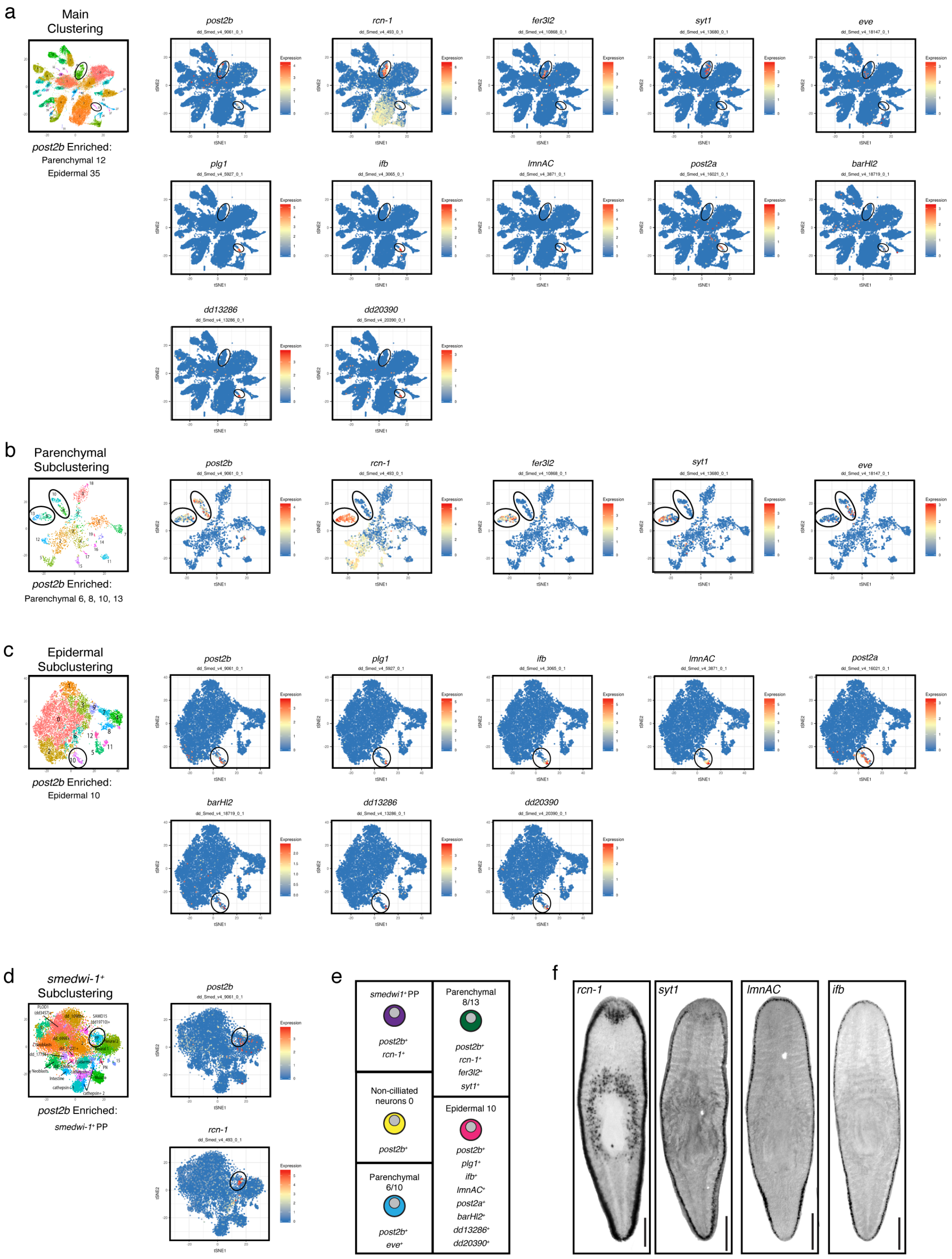

Supplementary Figure 9. Analysis of Hox downstream effector gene expression.
a-d t-SNE plots of *post2b* and effector gene expression data from (**a**) main clustering, (**b**) parenchymal sub clustering, (**c**) epidermal sub clustering, and (**d**) *smedwi-1⁺* sub

clustering from the cell type transcriptome atlas (Data sourced from Fincher et al., digiworm.mit.edu). Circles denote clusters with *post2b* enriched expression. **e** Summary of *post2b* and effector gene expression data from the cell type transcriptome atlas. **f** Expression of *post2b* gene effectors detected by colorimetric whole-mount in situ hybridization (n=4-7 animals, 2 independent repeats). Scale, 500um.

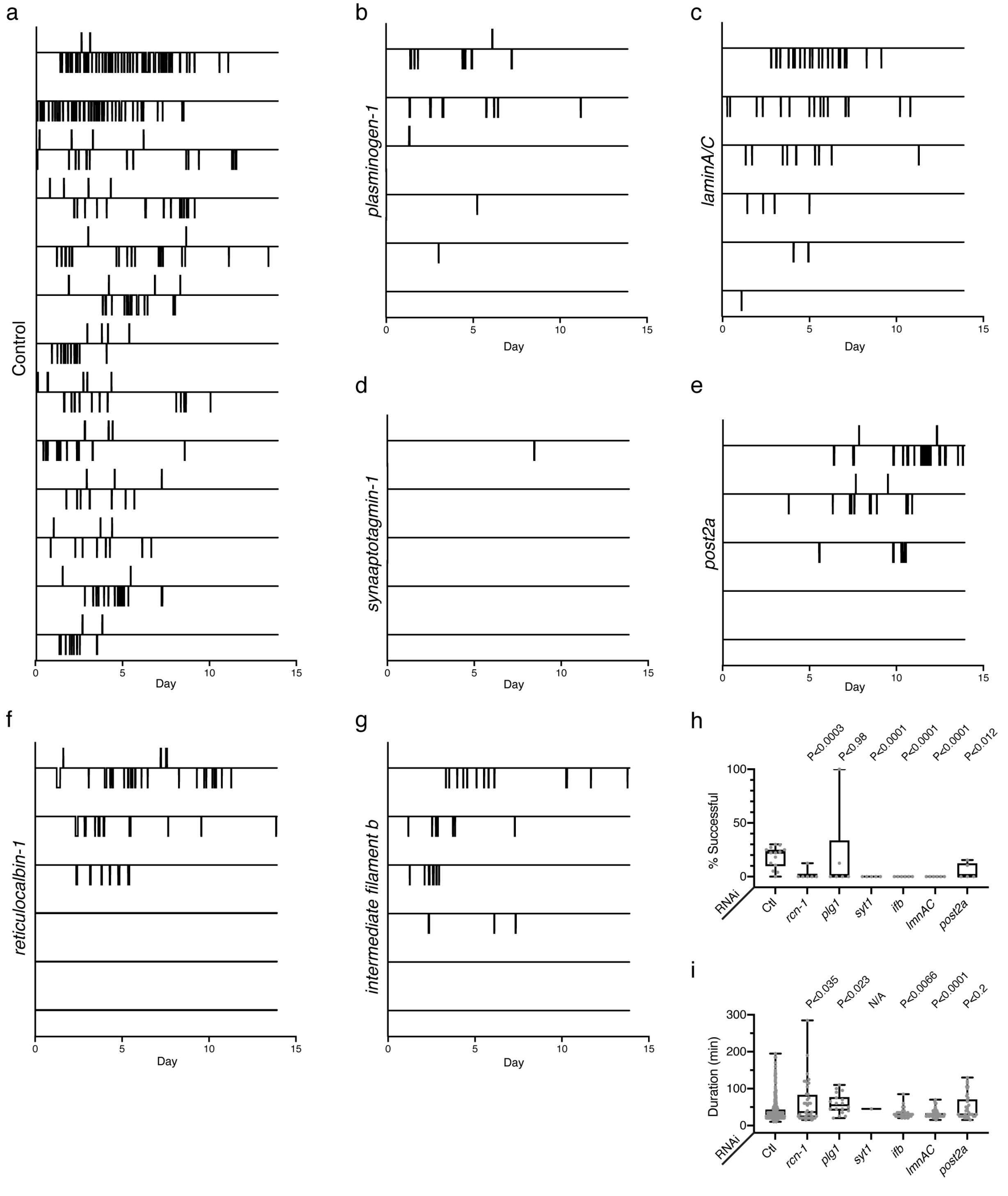

Supplementary Figure 10. ***post2b*** **downstream effectors regulate fission behavior.** **a-g** Fission activity timelines of (**a**) control, (**b**) *plasminogen-1*, (**c**) *laminA/C*, (**d**) *synaptotagmin-1*, (**e**) *post2a*, (**f**) *reticulocalbin-1*, and (**g**) *ifb* RNAi-treated animals. **h-i**

Plots of (**h**) percentage of successful attempts (Ctl vs. *rcn-1*, *plg1*, *syt1*, *ifb*, *lmnAC*, and *post2a* p-values=0.0002, 0.9742, <0.0001, <0.0001, <0.0001, and 0.0118, respectively) and (**i)** duration of each attempt (Ctl vs. *rcn-1*, *plg1*, *ifb*, *lmnAC*, and *post2a* p-values=0.0341, 0.022, 0.0066, <0.0001, and 0.1974, respectively) for RNAi treated animals. Animals fed bacteria expressing dsRNA 21 times (n= 14, 5, 5, and 6 animals for Ctl, *syt1*, *post2a*, and all other RNAi samples, respectively). Box and whisker plot depicts individual data points, median (centre), $25^{th}/75^{th}$ percentile (bounds of box), and minima/maxima (whiskers) P-values calculated by Welch's two-tailed t-test versus control. Source data are provided as a Source Data file.

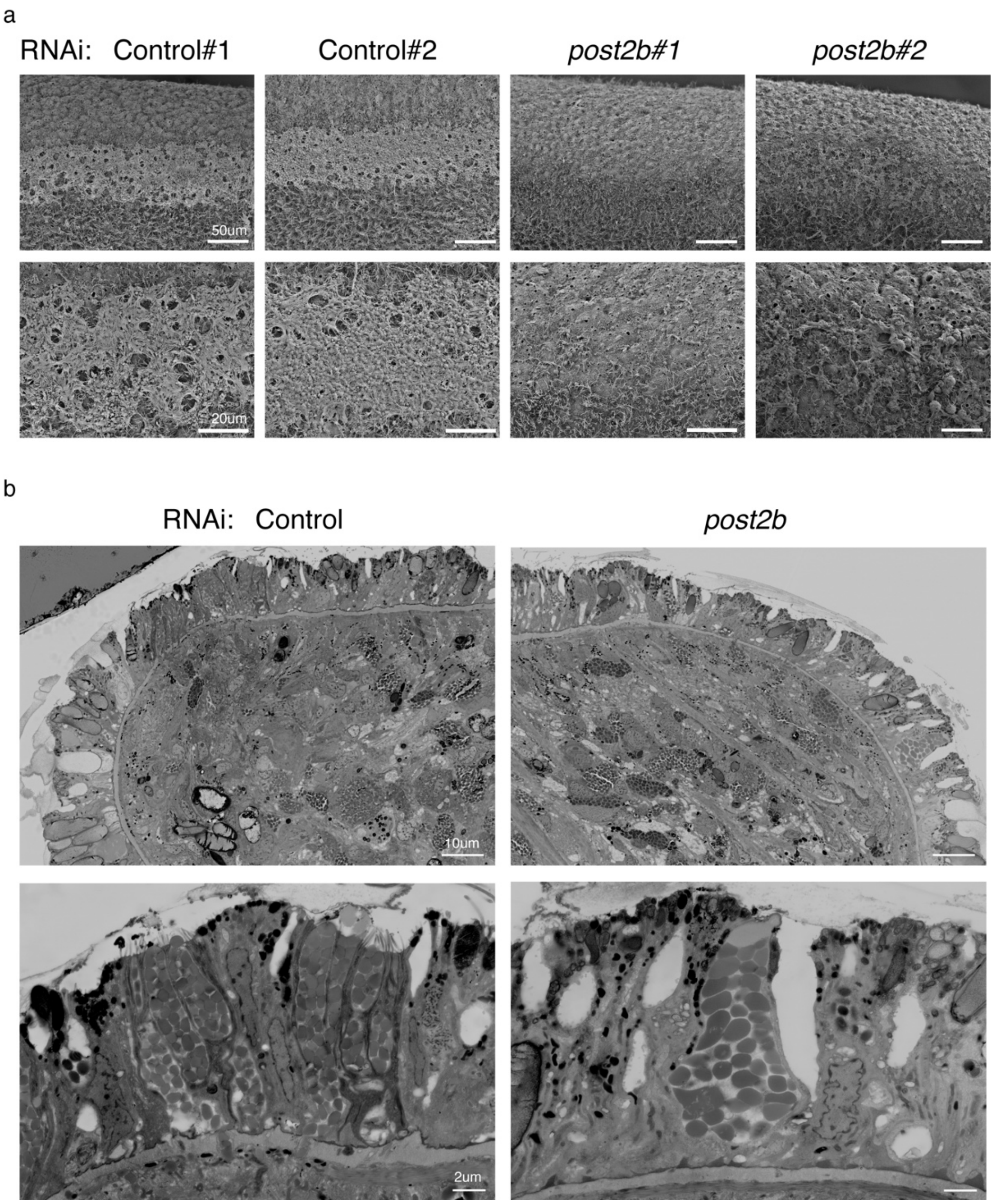

Supplementary Figure 11. *post2b* is required for the structure and functions of the marginal adhesive gland.

a Scanning electron micrographs of the lateral edge of the tail containing the marginal adhesive organ in control and *post2b* RNAi-treated animals. Examples of complete and partial phenotypic penetrance are depicted (n=3 animals, experiment independently repeated once). **b** Scanning electron micrographs of the marginal adhesive organ in

transverse sections in control and *post2b* RNAi-treated animals (n=3 animals, ventral up in all images, experiment not independently repeated).
