# Supplementary material for: Hox genes regulate asexual reproductive behavior and tissue segmentation in adult animals
Source: Nat Commun. 2021 Nov 18;12:6706. doi: 10.1038/s41467-021-26986-2 (PMC8602322; doi:10.1038/s41467-021-26986-2)
Supplement: Supplementary file 3 — Description of Additional Supplementary Files [file 41467_2021_26986_MOESM3_ESM.pdf]

Description of additional supplementary files

Title: Supplementary Data File 1.

Description: Results of Gene Expression Analysis of Hox RNAi RNAseq.

Title: Supplementary Data File 2.

Description: Summary of differential gene expression, scRNAseq analysis and Hox binding motif predictions for identified Hox downstream effectors.

Title: Supplementary Data File 3.

Description: Sequence identification and reference information and primers for genes listed in this study.

Title: Supplementary Data File 4.

Description: Statistical analyses performed in this report

Title: Supplementary Movie 1. Fission behavior in Control RNAi animals (Time-lapse A).

Description: Representative movie of fission behavior in control (*unc-22*) RNAi animals (8 purified dsRNA feedings). Worms were placed in 6-well dishes with cameras mounted above the plates and images acquired every 5 minutes for 15 days.

Title: Supplementary Movie 2.

Description: Fission behavior in *hox1* RNAi animals (Time-lapse A).

Representative movie of fission behavior in *hox1* RNAi animals (8 purified dsRNA feedings). Worms were placed in 6-well dishes with cameras mounted above the plates and images acquired every 5 minutes for 15 days.

Title: Supplementary Movie 3. Fission behavior in *hox3a* RNAi animals (Time-lapse A).

Description: Representative movie of fission behavior in *hox3a* RNAi animals (8 purified dsRNA feedings). Worms were placed in 6-well dishes with cameras mounted above the plates and images acquired every 5 minutes for 15 days.

Title: Supplementary Movie 4. Fission behavior in *hox3b* RNAi animals (Webcam Time-lapse A).
Description: Representative movie of fission behavior in *hox3b* RNAi animals (8 purified dsRNA feedings). Worms were placed in 6-well dishes with cameras mounted above the plates and images acquired every 5 minutes for 15 days.

Title: Supplementary Movie 5. Fission behavior in *lox5b* RNAi animals (Webcam Time-lapse A).
Description: Representative movie of fission behavior in *lox5b* RNAi animals (8 purified dsRNA feedings). Worms were placed in 6-well dishes with cameras mounted above the plates and images acquired every 5 minutes for 15 days.

Title: Supplementary Movie 6. Fission behavior in Control RNAi animals (Webcam Time-lapse B).
Description: Representative movie of fission behavior in control (*unc-22*) RNAi animals (17 RNAi feedings). Worms were placed in 6-well dishes with cameras below the plates and images acquired every 5 minutes for 14 days.

Title: Supplementary Movie 7. Fission behavior in *post2b* RNAi animals (Webcam Time-lapse B).
Description: Representative movie of fission behavior in *post2b* RNAi animals (17 RNAi feedings). Worms were placed in 6-well dishes with cameras below the plates and images acquired every 5 minutes for 14 days.

Title: Supplementary Movie 8. Fission behavior in *hox3a+hox3b* RNAi animals (Webcam Time-lapse B).

Description: Representative movie of fission behavior in *hox3a+hox3b* RNAi animals (17 RNAi feedings). Worms were placed in 6-well dishes with cameras below the plates and images acquired every 5 minutes for 14 days.

Title: Supplementary Movie 9. Phase I peristalsis in *hox3a+hox3b* RNAi animals.
Description: Representative movie of peristalsis after fission initiation in *hox3a+hox3b* RNAi animals (17 RNAi feedings). Images acquired at ten frames per second and played at 5X speed.

Title: Supplementary Movie 10. Phase I peristalsis in *hox3b* RNAi animals.
Description: Representative movie of peristalsis after fission initiation in *hox3b* RNAi animals (17 RNAi feedings). Images acquired at ten frames per second and played at 5X speed.

Title: Supplementary Movie 11. Fission behavior in Control RNAi animals (Webcam Time-lapse C).
Description: Representative movie of fission behavior in control (*unc-22*) RNAi animals (21 RNAi feedings). Worms were placed in 6-well dishes with cameras below the plates and images acquired every 5 minutes for 14 days.

Title: Supplementary Movie 12. Fission behavior in *reticulocalbin-1* RNAi animals (Webcam Time-lapse C).
Description: Representative movie of fission behavior in *reticulocalbin-1* RNAi animals (21 RNAi feedings). Worms were placed in 6-well dishes with cameras below the plates and images acquired every 5 minutes for 14 days.

Title: Supplementary Movie 13. Fission behavior in *plasminogen-1* RNAi animals (Webcam Time-lapse C).
Description: Representative movie of fission behavior in *plasminogen-1* RNAi animals (21 RNAi feedings). Worms were placed in 6-well dishes with cameras below the plates and images acquired every 5 minutes for 14 days.

Title: Supplementary Movie 14. Fission behavior in *synaptotagmin-1* RNAi animals (Webcam Time-lapse C).

Description: Representative movie of fission behavior in *synaptotagmin-1* RNAi animals (21 RNAi feedings). Worms were placed in 6-well dishes with cameras below the plates and images acquired every 5 minutes for 14 days.

Title: Supplementary Movie 15. Fission behavior in *intermediate filament b* RNAi animals (Webcam Time-lapse C).

Description: Representative movie of fission behavior in *intermediate filament b* RNAi animals (21 RNAi feedings). Worms were placed in 6-well dishes with cameras below the plates and images acquired every 5 minutes for 14 days.

Title: Supplementary Movie 16. Fission behavior in *laminA/C* RNAi animals (Webcam Time-lapse C).

Description: Representative movie of fission behavior in *laminA/C* RNAi animals (21 RNAi feedings). Worms were placed in 6-well dishes with cameras below the plates and images acquired every 5 minutes for 14 days.

Title: Supplementary Movie 17. Fission behavior in *post2a* RNAi animals (Webcam Time-lapse C).

Description: Representative movie of fission behavior in *post2a* RNAi animals (21 RNAi feedings). Worms were placed in 6-well dishes with cameras below the plates and images acquired every 5 minutes for 14 days.
