# Supplementary material for: Hox genes regulate asexual reproductive behavior and tissue segmentation in adult animals
Source: Nat Commun. 2021 Nov 18;12:6706. doi: 10.1038/s41467-021-26986-2 (PMC8602322; doi:10.1038/s41467-021-26986-2)
Supplement: Supplementary file 25 — Reporting Summary [file 41467_2021_26986_MOESM25_ESM.pdf]

nature research

Corresponding author(s): asa@stowers.org

Last updated by author(s): Aug 16, 2021

Reporting Summary

Nature Research wishes to improve the reproducibility of the work that we publish. This form provides structure for consistency and transparency in reporting. For further information on Nature Research policies, see our Editorial Policies and the Editorial Policy Checklist.

Statistics

For all statistical analyses, confirm that the following items are present in the figure legend, table legend, main text, or Methods section.

n/a	Confirmed	
☐	☒	The exact sample size (*n*) for each experimental group/condition, given as a discrete number and unit of measurement
☐	☒	A statement on whether measurements were taken from distinct samples or whether the same sample was measured repeatedly
☐	☒	The statistical test(s) used AND whether they are one- or two-sided<br>*Only common tests should be described solely by name; describe more complex techniques in the Methods section.*
☒	☐	A description of all covariates tested
☐	☒	A description of any assumptions or corrections, such as tests of normality and adjustment for multiple comparisons
☐	☒	A full description of the statistical parameters including central tendency (e.g. means) or other basic estimates (e.g. regression coefficient) AND variation (e.g. standard deviation) or associated estimates of uncertainty (e.g. confidence intervals)
☐	☒	For null hypothesis testing, the test statistic (e.g. *F*, *t*, *r*) with confidence intervals, effect sizes, degrees of freedom and *P* value noted<br>*Give P values as exact values whenever suitable.*
☒	☐	For Bayesian analysis, information on the choice of priors and Markov chain Monte Carlo settings
☒	☐	For hierarchical and complex designs, identification of the appropriate level for tests and full reporting of outcomes
☒	☐	Estimates of effect sizes (e.g. Cohen's *d*, Pearson's *r*), indicating how they were calculated

Our web collection on statistics for biologists contains articles on many of the points above.

Software and code

Policy information about availability of computer code

Data collection	The fission behavior timelapses were acquired using micro-manager version 1.4.22 (https://micro-manager.org/) and the opencvgrabber framework. In both cases, the camera gain, exposure, and autofocus were controlled using the Logitech Webcam Controller software (https://download01.logi.com/web/ftp/pub/video/lws/lws 280.exehttps://download01.logi.com/web/ftp/pub/video/lws/lws 280.exe).
Data analysis	Microsoft Excel (ver. 16.49), ImageJ (ver 2.1.0), and Graphpad Prism (ver.9.1) were used for Data Analysis.

For manuscripts utilizing custom algorithms or software that are central to the research but not yet described in published literature, software must be made available to editors and reviewers. We strongly encourage code deposition in a community repository (e.g. GitHub). See the Nature Research guidelines for submitting code & software for further information.

Data

Policy information about availability of data

All manuscripts must include a data availability statement. This statement should provide the following information, where applicable:

- Accession codes, unique identifiers, or web links for publicly available datasets
- A list of figures that have associated raw data
- A description of any restrictions on data availability

The original data generated in this study have been deposited in the Stowers Original Data Repository under accession code LIBPB-1567 [http://www.stowers.org/research/publications/LIBPB-1567]. The Hox RNAi RNAseq data generated from this study has been deposited in NCBI GEO with accession number: GSE159876 [https://www.ncbi.nlm.nih.gov/geo/query/acc.cgi?acc=GSE159876]. The fission progeny scoring data, compression and fission segment scoring data, fission progeny area calculations, fission behavior scoring data, and animal length measurements generated in this study are provided in the Source Data File. The RNAseq data for Supplementary Figures 2 and 3 used in this study are available in the NCBI GEO database under accession codes GSE82280 [https://www.ncbi.nlm.nih.gov/

geo/query/acc.cgi?acc=GSE82280] and GSE107874 [https://www.ncbi.nlm.nih.gov/geo/query/acc.cgi?acc=GSE107874]. The scRNAseq data used to generate t-SNE plots used in this study is available at https://digiworm.wi.mit.edu/ and NCBI GEO: GSE111764 [https://www.ncbi.nlm.nih.gov/geo/query/acc.cgi?acc=GSE111764]

Field-specific reporting

Please select the one below that is the best fit for your research. If you are not sure, read the appropriate sections before making your selection.

☒ Life sciences ☐ Behavioural & social sciences ☐ Ecological, evolutionary & environmental sciences

For a reference copy of the document with all sections, see nature.com/documents/nr-reporting-summary-flat.pdf

Life sciences study design

All studies must disclose on these points even when the disclosure is negative.

Sample size	Preliminary experiments suggested large effect sizes on fission rate following RNAi with many differences greater than 2-fold. Based on a power estimate, a sample size of six would give a 99% power with a 1% false positive rate. For Figure 1 we used a sample size ranging from 12 to 54 animals for our primary and secondary fission screens. For Figure 2 no sample size was calculation was performed, but we used a sample size ranging from 30-100 animals for compression and quantitation given the level of noise previously observed in this assay. For Figures 3, Supplementary Figure 5 and Supplementary Figure 6, a sample size of 5-8 replicates was used for live imaging due to technical protocol constraints. For Supplementary Figure For Figure 4i-j, a sample size of 3 animals was used for electron micrograph analysis due to limited RNAi-treated animal availability at that time. For other experiments, sample size was determined by the maximal number of animals that could be accommodated in accordance with the constraints of our animal husbandry, flow system, and technical protocols.
Data exclusions	Data points were omitted under the following pre-determined conditions: animal compression failed due to technical error, animals crawled out of wells and desiccated on lids during the fissioning experiment, and animals recently fissioned prior to recording of animal body length.
Replication	The number of independent repeats for the experiments has been labeled within the Figure Legends. All key findings were independently and successfully replicated at least once. Due to time constraints and sample availability Fig lb, 2d, 4g, 4hi-j, Supplementary Fig 4a, and Supplementary Figures 5, 6, 7, 10, 11 were performed only once.
Randomization	All organisms were size-matched and randomly distributed into experimental treatments
Blinding	Investigators were single-blinded via coded numbers to obfuscate the identity of genes targeted by RNAi for data collection and analysis.

Reporting for specific materials, systems and methods

We require information from authors about some types of materials, experimental systems and methods used in many studies. Here, indicate whether each material, system or method listed is relevant to your study. If you are not sure if a list item applies to your research, read the appropriate section before selecting a response.

Materials & experimental systems

n/a	Involved in the study	
☐	☒	Antibodies
☒	☐	Eukaryotic cell lines
☒	☐	Palaeontology and archaeology
☐	☒	Animals and other organisms
☒	☐	Human research participants
☒	☐	Clinical data
☒	☐	Dual use research of concern

Methods

n/a	Involved in the study	
☒	☐	ChIP-seq
☒	☐	Flow cytometry
☒	☐	MRI-based neuroimaging

Antibodies

Antibodies used	Anti-Fluorescein-POD (Roche #11426346910), Anti-Digoxigenin-POD (Roche #11207733910), and Anti-Digoxigenin-AP (Roche #11093274910).
Validation	No validation of these antibodies in this specific planarian species was performed. Literature describing their common use in the planarian field is cited.

Animals and other organisms

Policy information about studies involving animals; ARRIVE guidelines recommended for reporting animal research

Laboratory animals	The clonal CIW4 strain of Schmidtea mediterranea maintained in recirculatory culture were used for all experiments. These animals are immortal asexual adults.

Wild animals	Study did not involve wild animals.
Field-collected samples	Study did not involve samples collected from the field
Ethics oversight	Husbandry and experimental protocols for invertebrate planarians are not subject to ethical approval via IACUC.

Note that full information on the approval of the study protocol must also be provided in the manuscript.
